# GC-MS Based Metabolite Profiling to Monitor Ripening-Specific Metabolites in Pineapple (*Ananas comosus*)

**DOI:** 10.3390/metabo10040134

**Published:** 2020-03-31

**Authors:** Muhammad Maulana Malikul Ikram, Sobir Ridwani, Sastia Prama Putri, Eiichiro Fukusaki

**Affiliations:** 1 Department of Biotechnology, Graduate School of Engineering, Osaka University, 2-1 Yamadaoka, Suita, Osaka 565-0871, Japan; malikul_ikram@bio.eng.osaka-u.ac.jp (M.M.M.I.); fukusaki@bio.eng.osaka-u.ac.jp (E.F.); 2 Center for Tropical Horticulture Studies, IPB University, Jl. Baranangsiang, Bogor 16144, Indonesia; ridwanisobir@gmail.com

**Keywords:** pineapple, metabolomics, ripening, non-climacteric

## Abstract

Pineapple is one of the most cultivated tropical, non-climacteric fruits in the world due to its high market value and production volume. Since non-climacteric fruits do not ripen after harvest, the ripening stage at the time of harvest is an important factor that determines sensory quality and shelf life. The objective of this research was to investigate metabolite changes in the pineapple ripening process by metabolite profiling approach. Pineapple (Queen variety) samples from Indonesia were subjected to GC-MS analysis. A total of 56, 47, and 54 metabolites were annotated from the crown, flesh, and peel parts, respectively. From the principal component analysis (PCA) plot, separation of samples based on ripening stages from C0–C2 (early ripening stages) and C3–C4 (late ripening stages) was observed for flesh and peel parts, whereas no clear separation was seen for the crown part. Furthermore, orthogonal projection to latent structures (OPLS) analysis suggested metabolites that were associated with the ripening stages in flesh and peel parts of pineapple. This study indicated potentially important metabolites that are correlated to the ripening of pineapple that would provide a basis for further study on pineapple ripening process.

## 1. Introduction

**Figure 1:**
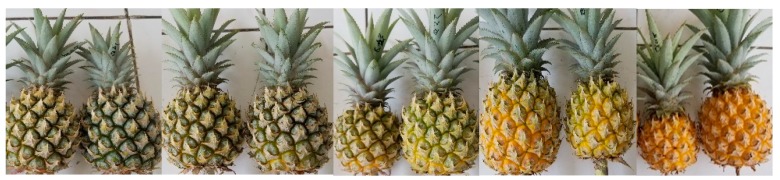
Pineapple sample from all ripening stages. From left to right: C0 until C4 stages. This classification is based on the peel color of pineapple, in which C0 stage contains 0% yellow color, C1 stage contain 0%–25% yellow color, C2 stage contain 25%–50% yellow color, C3 stage contain 50%–75% yellow color, and C4 stage contain 75%–100% yellow color [5].

Pineapple (*Ananas comosus*) market value is approximately 14.7 billion USD with a production volume of around 25 million metric tons in the world [1]. Pineapple is categorized as a non-climacteric fruit. The major difference between climacteric and non-climacteric fruit is non-climacteric fruit produces low levels of ethylene and does not show any major peak in the respiration rate during the ripening process, whereas climacteric fruit depends on ethylene bursts during ripening [2,3]. In addition, ethylene treatment does not give any effect to non-climacteric fruit with the exception of degreening (removal of chlorophyll) [4]. Another distinct characteristic of non-climacteric fruit is the fruit will not continue its ripening process after harvest, thus making it important to be harvested in the right ripening stage to ensure proper quality [3]. The ripening stage of pineapple is divided into 5 stages, C0–C4, with the green-ripe fruit at C0 and the full-ripe fruit at C4 based on United Nations Economic Commission for Europe (UNECE) Standard for pineapple (FFV-49) as seen in Figure 1 [5]. This classification is based on the peel color of pineapple, in which C0 stage contains 0% yellow color, C1 stage contain 0%–25% yellow color, C2 stage contain 25%–50% yellow color, C3 stage contain 50%–75% yellow color, and C4 stage contain 75%–100% yellow color [5]. Pineapple is usually exported in the C1 stage [6], while the fully ripe fruit (C4 stage) is mainly for domestic consumption. The fruit of pineapple consists of the fusion of individual fruits. These individual fruits are developed from a single flower and the external of these fruits were protected with a hard-polygonal shield, commonly called as pineapple peel [7]. On top of pineapple fruit, there are leaves that can be used for vegetative reproduction of pineapple commonly called the pineapple crown. This crown part is commonly harvested along with the fruit harvest [7]. Crown and peel parts develop during pineapple fruit development. Therefore, to understand the pineapple ripening process comprehensively, analysis of pineapple peel and crown is needed in addition to flesh analysis. At present, there is limited information on the differences in metabolite composition of pineapple from different ripening stages [8]. Monitoring metabolites changes using tools such as metabolomics, a comprehensive study of metabolite, is a powerful tool for further understanding pineapple ripening process.

Recent studies about fruit ripening metabolomics mostly focused on climacteric fruits, such as banana, mango, capsicum, dates, avocado, peach, climacteric melon, and mangosteen [9,10,11,12,13,14,15,16]. On the other hand, only a few of non-climacteric ripening processed fruit had been elucidated using metabolomics approach, such as cherry, blackcurrant, blueberry, non-climacteric melon, and pineapple [15,17,18,19,20,21]. Previous metabolomics studies on non-climacteric fruit employed mass spectrometry-based instruments, such as gas chromatography-mass spectrometer (GC-MS) or liquid chromatography-mass spectrometer (LC-MS). Several studies performed a combination of headspace-solid phase microextraction (HS-SPME) with GC-MS to measure volatile compounds during the ripening process [15,20]. Reports on pineapple ripening have been focused in volatile and phenolic compounds using HS-SPME-GC-MS, high-performance liquid chromatography with diode array detection and electrospray ionization multiple-stage mass-spectrometry (HPLC-DAD-ESI-MS^n^), and electrospray ionization mass spectrometry (ESI(-)FT-ICR MS) [21,22,23,24]. The outcome from these previous reports suggested the changes in phenolic patterns, such as coumaroyl isocitrate and *S-p*-coumaryl, and volatile compounds, such as methyl 3-(methylthio)propanoate and δ-octalactone, along the pineapple ripening process.

As mentioned previously, previous studies on pineapple ripening were on the targeted analysis of volatile and phenolic compounds using the flesh part as a sample. To date, there is no study that analyzed different parts of pineapple including flesh, peel, and crown parts, and incorporating broad coverage of primary metabolites such as sugar, organic acid, amino acid, sugar alcohol, sugar acid, and amine compounds. In order to suggest metabolites that are associated with ripening, there are several different multivariate analyses that can be used. The most common multivariate analysis is principal component analysis (PCA) and orthogonal projections to latent structures (OPLS) regression analysis [25,26]. In this study, a metabolite profiling approach using GC-MS in combination with PCA and OPLS was conducted to monitor the changes of primary metabolites (sugar, organic acid, amino acid, etc.) during pineapple ripening process by analyzing pineapple fruit (crown, flesh, and peel) from different ripening stage (Figure S1). OPLS model was constructed using metabolites annotated by GC-MS as an explanatory variable and ripening stages as a response variable. The constructed model from flesh and peel samples indicated several potentially important metabolites that were correlated with the pineapple ripening process. This study would be important to complement the knowledge of the pineapple ripening process and could serve as a basis for post-harvest handling strategy in pineapple industries.

## 2. Results

### 2.1. Optimization of Sample Preparation Methods in Pineapple Fruit

The first analysis was to compare two different sample preparation methods, namely food processor and freeze-drying. This analysis was conducted to optimize sample preparation of pineapple fruit in GC-MS analysis. A total of 47 metabolites belonging to various metabolite classes were annotated by GC-MS analysis in flesh samples (Table S1). These metabolites comprise of 26 metabolites belongs to sugars class, 12 metabolites belong to amino acids and amines, and 9 metabolites belong to organic acids. These annotated metabolites were subjected to PCA to clearly visualize the differences in metabolite levels in pineapple flesh prepared by food processing and freeze-drying methods.

**Figure 2:**
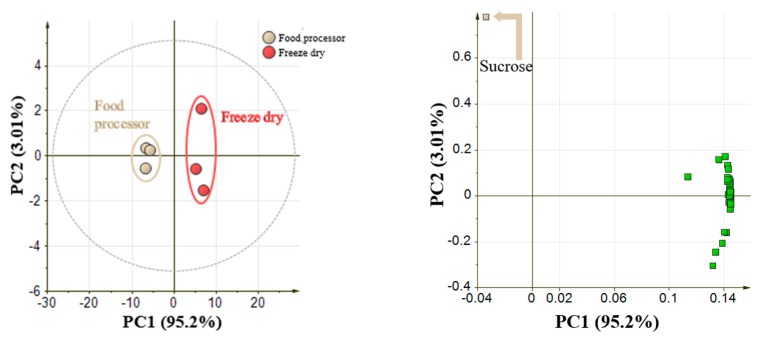
PCA of comparison of different sample preparation methods: Food processor method and freeze-drying method. 47 annotated metabolites from GC-MS analysis were auto-scaled prior to PCA. (Left: Score plot between food processor and freeze dry samples; Legends represent the samples and colored as follow: food processor: light brown circle, freeze dry: red circle. Right: Loading plot shown almost all metabolites (except sucrose shown by light brown arrow) showed higher accumulation by freeze-dry method).

**Figure 3:**
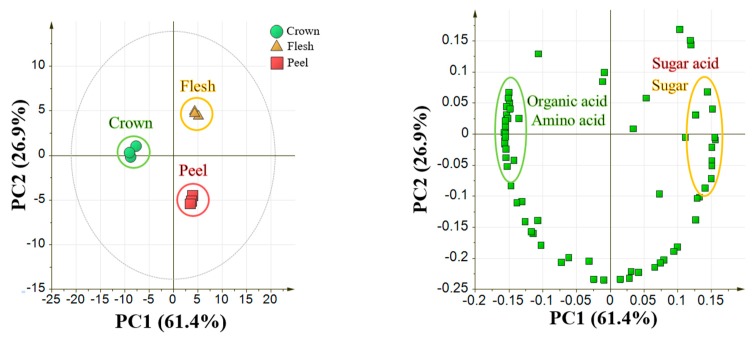
PCA result from different parts of pineapple. 64 tentatively identified metabolites from GC-MS analysis were auto-scaled prior to PCA. Left: Score plot between crown, flesh, and peel part. Legends represent the sample and colored as follows: crown: green circle, flesh: yellow triangle, peel: red square. Right: Loading plot shown that crown part accumulates organic and amino acid, while flesh and peel accumulate sugar and sugar acid.

Figure 2 shows the comparison between these two methods after analysis by GC-MS. From the score plot, flesh samples prepared by two different methods were clearly separated along the PC1 with a 95.2% variance. The loading plot showed that almost all metabolites were accumulated in the flesh samples prepared by freeze-dry method. Only sucrose was found to accumulate in samples prepared by food processor method. In the next analysis, we applied the freeze-drying method to analyze different parts of pineapple in GC-MS. A total of 54, 44, and 50 metabolites were annotated from crown, flesh, and peel, respectively (Table S2). PCA score plot from Figure 3 clustered pineapple fruit into three different parts (crown, flesh, and peel) based on the metabolite distributions. Amino acid and organic acid were found to be accumulated in crown part, while the peel and flesh part show accumulation of sugar and sugar-acid. Due to the separation for each part, this result becomes the basis to analyze three parts of pineapple separately.

### 2.2. GC-MS and Principal Component Analysis of Pineapple from Different Ripening Stages

Analysis of crown, flesh, and peel parts of pineapple from different ripening stages was conducted separately. Metabolite profiling approach using GC-MS instrument could detect 351 metabolite peaks in the crown part, 297 metabolite peaks in the flesh part, and 359 metabolite peaks in the peel part. Among those peaks, 85 peaks in the crown part, 74 peaks in the flesh part, and 73 peaks in the peel part were annotated using MSP Library containing RI and EI-MS from our laboratory experimental data. Metabolites from QC samples with RSD more than 20% were excluded from the analysis [27]. After exclusion of metabolites with RSD higher than 20%, the number of annotated metabolites in crown part were 56 metabolites, in the flesh part were 47 metabolites, and in the peel part were 54 metabolites. A complete list of these metabolites during ripening analysis is shown in Table S3.

**Figure 4:**
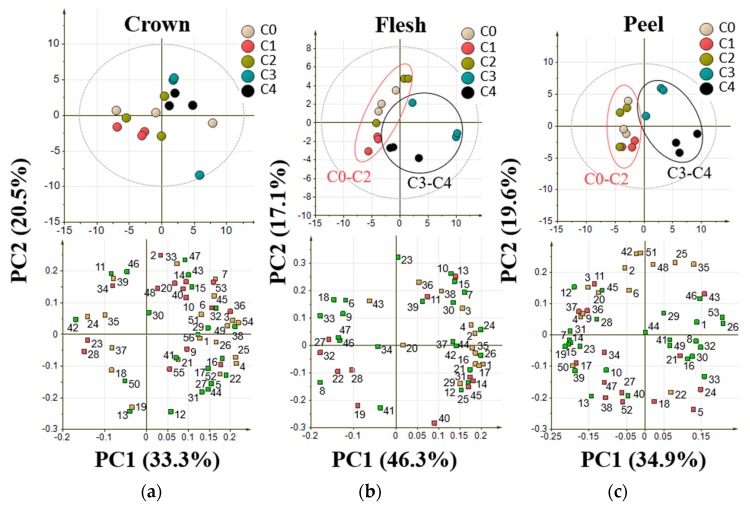
PCA result from flesh, crown and peel parts of pineapple from different ripening stages. Variables used for PCA were 56, 47, and 54 annotated metabolites by GC-MS from crown, flesh, and peel parts respectively. Data was auto scaled prior to PCA. (**a**) Score and loading plot from the crown part; (**b**) score and loading plot from the flesh part; (**c**) score and loading plot from the peel part. Legends represent the samples and colored as follows: brown: C0 stage, red: C1 stage, green: C2 stage, blue: C3 stage, black: C4 stage. Upper part show score plot; Bottom part show loading plot. Loading plot was colored based on metabolite classes: green: sugars; red: organic acids; yellow: amino acids and amines.

Figure 4 shows the score plot from PCA for pineapple from different ripening stages (C0 to C4 stage) as observed in three different parts of pineapple. As seen in Figure 4b,c, flesh and peel part showed two distinct clusters along PC1. Less ripe samples (C0–C2) were clustered together and ripe fruit samples (C3 and C4) formed a separate cluster. This trend was explained by 63.9% and 53.3% variance in the flesh and peel part, respectively. However, this trend was not shown in the crown part of pineapple from all principal components. Loading plot in Figure 4b,c showed the metabolite accumulation in the less ripe and ripe samples for flesh and peel part, respectively. These metabolite intensities that used to create a score plot were normalized using an internal standard, ribitol. The internal standard was chosen because it is not present in pineapple samples and stable in a mixed solvent solution.

### 2.3. Orthogonal Projection to Latent Structures of Pineapple Ripening Process

**Figure 5:**
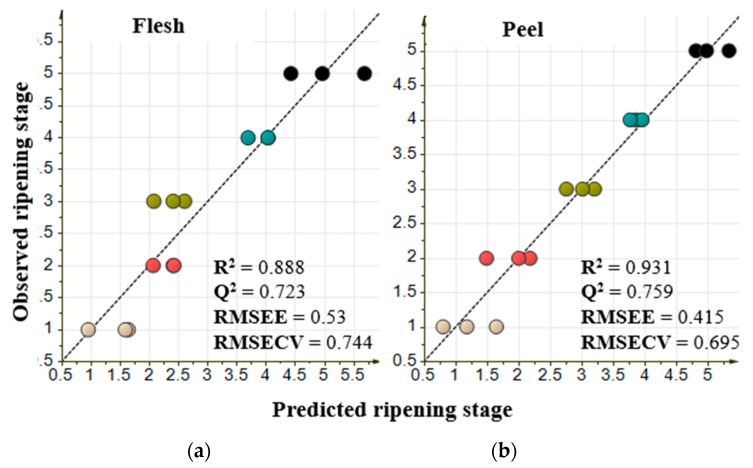
Orthogonal projection to latent structures (OPLS) results from flesh and part of pineapple. Explanatory variables in flesh part are 47 metabolites, while in peel part are 54 metabolites. Response variable for both models is the ripening stages with numbered as follows: C0 stage as 1, C1 stage as 2, C2 stage as 3, C3 stage as 4, and C4 stage as 5. Value of R^2^, Q^2^, RMSEE, and RMSECV were used to evaluate the model. (**a**) OPLS model of flesh part; (**b**) OPLS model of peel part.

Orthogonal projection of latent structures (OPLS) regression analysis was conducted to identify metabolites that were highly influenced by the process related to the response variable [28]. In this study, two latent variables were used to construct the model using flesh and peel parts of pineapple. Crown part was not analyzed based on the previous result in PCA which indicated that crown part was not able to show any trend in ripening process. Response variables that were used to generate the model were ripening stages from C0 as 1, C1 as 2, C2 as 3, C3 as 4, and C4 as stages 5, while the explanatory variables were metabolites annotated by GC-MS analysis. Pineapple from C0 to C4 ripening stages harvested in April 2019 were used as a training set to generate the model (Figure 5). Model validation was conducted by leave-one-out cross-validation from each replicate.

**Figure 6:**
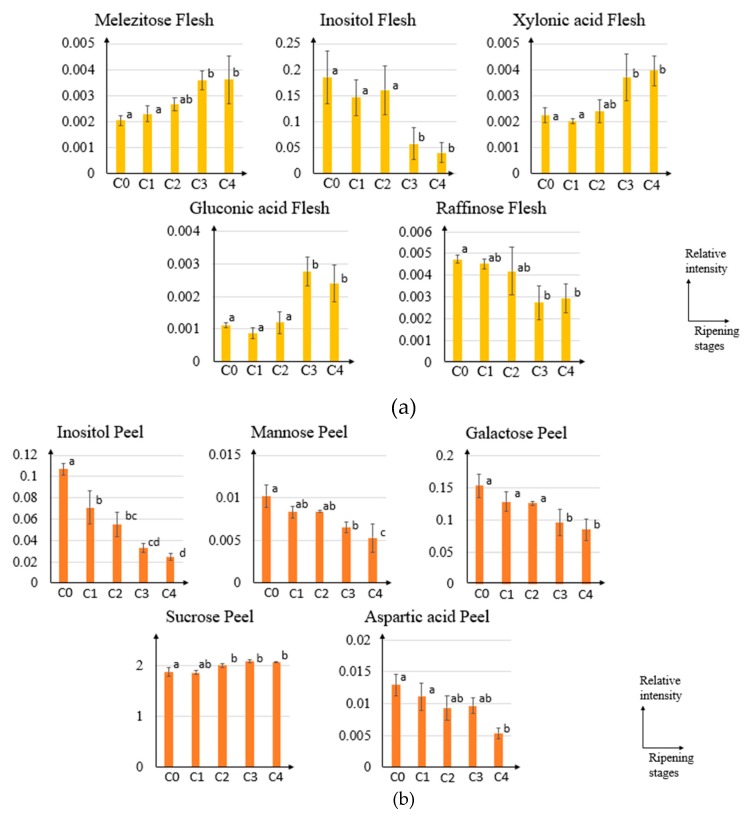
Bar graph of five highest variable important in projection (VIP) metabolites related to pineapple ripening process in (**a**) flesh and (**b**) peel part. The relative intensity of the five highest VIP metabolites was normalized by the internal standard. Vertical axis represents metabolites relative intensity and horizontal axis represents ripening stages. Significant differences (*p* < 0.05) are indicated with the different letters based on mean comparison Tukey’s test.

The constructed OPLS regression model with R^2^ of 0.888 and 0.931 for flesh and peel part, respectively, are shown in Figure 5. In the OPLS regression analysis, statistically important metabolites for the models were indicated by the score of variable important in projection (VIP). Metabolites with a VIP score of more than 1 considered important for the model [28] (Table S4). Contributing metabolites were chosen based on the five highest VIP scores. The metabolites were melezitose, inositol, xylonic acid, gluconic acid, and raffinose in the flesh model, whereas inositol, mannose, galactose, sucrose, and aspartic acid were the top five highest VIP metabolites in the peel. Among these highest VIP metabolites in both flesh and peel, melezitose, xylonic acid, gluconic acid, and sucrose have a positive correlation with the ripening stages, while inositol, raffinose, mannose, galactose, and aspartic acid showed a negative correlation with the ripening process (Figure 6).

## 3. Discussion

Metabolite profiling is known to be useful to analyze a large group of metabolites that belong to a specific class of compounds that reflects the dynamic response to physiological change or developmental stimuli [29,30] In this study, a metabolite profiling approach using GC-MS was employed for the study of pineapple ripening process. GC-MS is suitable for metabolite profiling because it provides high sensitivity, reproducibility, and quantitation of a large number of metabolites with a single-step extraction [31,32]. Metabolite annotated in pineapple crown, peel and flesh were classified as sugars, amino acids, amines, organic acids, and other compounds. Sugars were found to be the most abundant in pineapple. This is in agreement with previous work that mentioned the high content of sugars was observed in pineapple flesh samples [21]. In this study, we conducted for the first time the analysis of peel and crown parts of pineapple in addition to flesh samples. Annotated metabolites from each part were subjected to PCA and OPLS analyses. Principal component analysis (PCA) is a multivariate data analysis that could show the variance among the samples using metabolites as the explanatory data [33]. Based on the PCA, pineapple ripening was clustered into two major phases namely C0-C2 stages (early ripening) and C3-C4 stages (late ripening). The trends above were observed only in flesh and peel samples, whereas there was no clear trend of ripening in crown part. It was previously suggested that crown photo-assimilation seems to be derived from its own photosynthesis, not from the fruit [34]. It is also well known that pineapple maturation developed from the bottom, not from the top [35]. Therefore, crown part is considered to have no correlation with fruit ripening.

Further analysis to identify potentially important metabolites that are correlated with the ripening process was conducted using OPLS regression analysis. OPLS regression analysis is known to be more powerful to explain the relationship between the response variable and explanatory variable because it is a supervised multivariate analysis [28]. The OPLS regression model shown in Figure 5 has some parameters that could be used to evaluate the quality of the model itself. These parameters are R^2^, Q^2^, RMSEE, and RMSECV. R^2^ is defined as the square of the correlation coefficient between observed and predicted value in a regression [36]. Q^2^ is known to be a reliable parameter for model predictivity [16,36]. RMSEE or root mean square error of estimation and RMSECV or root mean square error of cross-validation are the values to evaluate accuracy, prediction, and model robustness [36,37]. A good model would have an R^2^ value of more than 0.6, Q^2^ value of more than 0.6, and a low value of RMSEE and RMSECV [36]. We constructed 3 OPLS models from metabolites annotated in flesh, peel and crown. The constructed model of flesh and peel showed R^2^ value of more than 0.6, Q^2^ value of more than 0.6, and a low value of RMSEE and RMSECV, whereas crown model showed R^2^ value of 0.896, Q^2^ value of 0.432, RMSEE value of 0.509, and RMSECV value of 1.066. These results indicated that only peel and flesh model meet the thresholds for a valid model with a good fit. The low Q^2^ value in crown model showed that the samples from crown part cannot be used to predict ripening stages in pineapple. This is in line with the results obtained from PCA.

Contributing metabolites related to ripening stages could be obtained from the variable importance in the projection (VIP) scores. Based on these scores, the five highest VIP metabolites in the flesh part are melezitose, inositol, xylonic acid, gluconic acid, and raffinose; while for peel part are inositol, mannose, galactose, sucrose, and aspartic acid. Figure 6 shows the dynamic of these VIP metabolites relative intensity (normalized with ribitol) along the ripening process of pineapple. These metabolites were shown to be increased or decreased during the pineapple ripening process. From these VIP metabolites in flesh parts, the raffinose level was in agreement with the previous report that showed a decreased level during ripening process [38]. In addition to the previously reported metabolites that correlate with the ripening process, this study also reports the dynamics of inositol, melezitose, xylonic acid, and gluconic acid in the flesh part during ripening process. Inositol or commonly known as myo-inositol was known to regulate osmotic pressure in blueberry fruit thus maintaining turgor and fruit firmness between the firm cultivar and soft cultivar [19]. In addition to that, inositol might be oxidized to D-glucuronic acid known as a major precursor of the cell wall in Arabidopsis [39]. Therefore, the presence of inositol might also relate to the cell wall in fruit. Melezitose is known to play a role in osmoregulation system [40]. However, comparing the relative intensity trend with inositol, the mechanism underlying these two metabolites might be different to regulate the osmoregulation system during the pineapple ripening process. Even though melezitose relative intensity was considered low compared to other sugar, it was reported in the previous study that it could attract ants in honeydew fruit [41]. Therefore, the accumulation of melezitose in the latter stage of ripening might reflect the attractancy of the fruit in the fully ripe stage.

Xylonic acid relative intensity was shown to be increased during the ripening process (Figure 6a). The increase of this organic acid might be related to the reactive oxygen species (ROS). During fruit ripening, oxidative stress was increased and might result in some changes in fruit, such as changes in skin color or fruit softening. Due to the presence of ROS, fruit antioxidants might act to balance the reduction–oxidation homeostasis [42]. One of the most known fruit antioxidants is ascorbic acid. The previous report stated that xylonic acid is a product of ascorbic acid degradation, thus explaining the increase of xylonic acid during the ripening process [43]. During the pineapple ripening process, raffinose was found to be decreased along with the progression of ripening (Figure 6). In agreement with this result, previous report showed that the raffinose level also decreases in Japanese plum non-climacteric cultivar during its ripening [38]. They reported that the raffinose level in non-climacteric fruit might be related to its ability to alleviate the oxidative process during fruit ripening [38]. Therefore, not only xylonic acid but the level of raffinose might also be related to the reduction–oxidation process along the ripening process. The gluconic acid concentration was shown to be increased during the late-ripening process in pineapple (Figure 6a). This increase might be triggered by the increase of carbon molecules availability during the later stage of pineapple ripening [44]. In addition, gluconic acid intensity increase might also cause by the effect of cell wall degradation, change in cuticle composition and pH of host cells that allow the transition of fungi into their aggressive colonization [44]. The presence of gluconic acid might indicate an infection that could secrets gluconic acid and acidify the pH in fruit such as in apple and mango [45,46].

Metabolite that shows the highest VIP score in peel after inositol is mannose. Mannose is known as a component of the plant cell wall, specifically hemicellulose [47]. The previous report mentioned that the concentration of mannose was decreased during fruit development, hence support our findings shown in Figure 6b [48]. Similar to mannose, decrease level of aspartic acid in the pineapple ripening process were also reported during ripening of banana [49]. Aspartic acid was known to be a source for umami taste along with glutamic acid [50]. Its level was known to be varied among the fruits. In banana and *Vasconcellea quercifolia*, the level of aspartic acid was found to be decreased along with the ripening progress, while mature or ripe tomato contained more aspartic acid that brings out the umami taste [49,51,52]. Other than the source of umami taste, the level of aspartic acid might also connect with the free auxin level in fruits that affect its ripening [53]. Aspartic acid was found to be conjugated with indole acetic acid (IAA) and lead to degradation of the IAA hormones [53].

Other than the previously reported metabolites, this study found the change of sucrose and galactose level in the peel part might correlate with the pineapple ripening process. This study showed the sucrose level in pineapple peel part was increased during ripening (Figure 6b) while previous report mentioned the increase of sucrose during ripening process in pineapple flesh [21]. Sucrose was known to become a source of sweet taste in food and commonly used as a standard solution for sweetness [54]. However, sucrose in the peel part might not directly affect the sweetness in the flesh. It is reported that sucrose in the peel is lower if compared with the flesh part [55]. Sucrose not only contributes to sweetness, but it also plays a role to regulate fruit development and ripening in strawberry fruit, a non-climacteric fruit [56]. Sucrose accumulation might induce the expression level of the key enzymes in abscisic acid (ABA) hormones pathway, hence promote ripening process in non-climacteric fruit via ABA hormones [57]. Galactose in Figure 6b was shown a decreasing trend along the ripening stages. This might be explained by the relation of galactose with the plant cell wall. The previous report showed that galactose is the major non-cellulosic sugar in the cell wall and significantly decreased during fruit ripening [58,59]. All these metabolites with high VIP score consist of sugars (melezitose, inositol, raffinose, mannose, galactose, and sucrose) and organic acids (xylonic acid, gluconic acid, and aspartic acid).

During ripening process, many biological processes occur, such as cell wall loosening, texture changes, flavor development, chlorophyll degradation, and pigment accumulation [60]. Changes in melezitose and inositol level might indicate the texture changes during pineapple ripening process. Melezitose and inositol are known to regulate fruit firmness that affects the texture or hardness of fruit [19]. In addition to that, inositol, galactose, and mannose levels might be related with cell wall loosening during ripening. Inositol were known to be precursor of D-glucuronic acid of plant cell wall, galactose is a major non-cellulosic sugar in plant cell wall, and mannose are component of hemicellulose in plant cell wall. Therefore, the decrease of these three metabolites might correlate with loosening of cell wall that usually accompanied with decrease level of firmness and increase of gluconic acid [19,44]. Reactive oxygen species also play a role in ripening process to regulate programed cell death and cell aging [61]. This report in line with our results that show decrease level of xylonic acid and increase level of raffinose during ripening process. Both metabolites were known to respond to reactive oxygen species as discussed previously. These biological processes that affected by the ripening process are product of biochemical changes that mediated by plant hormone. Abscisic acid (ABA) and auxin were known to be affecting the ripening process in non-climacteric fruit [60]. Changes in sucrose and aspartic acid during ripening process might affected abscisic acid and auxin, respectively. Therefore, it might modulate ripening process in pineapple fruit.

This study showed the significance of sample preparation to gain more metabolite coverage that is useful for further analysis. Based on metabolites data acquired from GC-MS analysis, flesh and peel data could show clustering separation between C0-C2 stages and C3-C4 stages using principal component analysis (PCA), while the crown part does not show correlation with the ripening process. Orthogonal projection to latent structures (OPLS) regression analysis reveals metabolites that have possible relations to the pineapple ripening process in flesh and peel parts. In the flesh part, melezitose, inositol, xylonic acid, gluconic acid, and raffinose were found to be the five highest important metabolites, while for the peel part are inositol, mannose, galactose, sucrose, aspartic acid. These metabolites were known to be involved during plant cell wall metabolism and osmoregulation system thus affecting the firmness and shelf life of pineapple, in addition to the redox defense system and non-climacteric ripening hormones. For future applications, these VIP metabolites could be added exogenously to regulate specific effects, for example, the addition of polyamine and ascorbic acid to regulate the shelf life of fruit, as shown in the previous reports [62,63]. In addition, influencing the level of the metabolites through post-harvest treatment was also feasible to be conducted, such as regulating inositol, galactose, and raffinose by cold or heat treatment [64,65,66]. It must be noted that this study only limited to the “Queen” cultivar. Future study using other widely known cultivars, such as “Smooth Cayenne”, is still needed to enrich the information regarding pineapple ripening process. Regardless, this study might become a basis for resolving the post-harvest issue in the pineapple industry by controlling important metabolites influenced in the ripening process.

## 4. Materials and Methods 

### 4.1. Plant Materials

Pineapple (*Ananas comosus*) fruit from Indonesia corresponding to 5 different ripening stages were used in this study (Figure 1). To set the same harvest time at the end of April 2019, ethephon treatment was used to induce fruit development around November to December 2018. Cultivars of pineapple used in this study to represent the important cultivar from the pineapple Industry is cv. Mahkota Bogor ‘Queen’. Three samples (biological replicates) from different plants were collected from each ripening stage for Queen cultivars from the cultivation period of November 2018—April 2019 in Center for Tropical Horticulture Studies, Bogor Agricultural University (CENTROHS, Bogor Agriculture University), Bogor, Indonesia (minimum temperature 21 °C and maximum temperature 35 °C). Ripening stage determination was conducted with the help of a trained panelist according to peel color changes [5].

### 4.2. Optimization of Sample Preparation

#### 4.2.1. Comparison between Food Processor and Freeze-Drying Methods

Pineapple fruit was cut into half then the flesh was diced using a stainless-steel knife. Diced flesh with 1 × 1 cm size from one half of fruit was subjected to freeze dry-extraction method using VD-800F Freeze dryer (Taitec, Saitama, Japan). This flesh was put into a Pyrex tube and closed with holed parafilm before being quenched with liquid nitrogen and lyophilized. Diced flesh with 1 × 1 cm size from the other half of the fruit was homogenized using hand immersion-blender WSB-33XJ (Waring Commercial, Pennsylvania, United States of America) before extraction. Ten milligrams of both samples were subjected to extraction following the method described previously prior to GC-MS analysis [66].

#### 4.2.2. Comparison of Crown, Flesh, and Peel of Pineapple Fruit

The pineapple was cut into three different parts, crown, flesh, and peel. Crown part was analyzed by cutting the leaves into a 1x1 cm size before quenching by liquid nitrogen and freeze-dried. Peel part was analyzed by scraping the peel into a 1x1 cm size using a stainless-steel knife before quenching and freeze-drying. Flesh part was analyzed according to above description. Lyophilized sample was homogenized using Multi-beads shocker (Yasui Kikai, Osaka, Japan). Ten milligrams of the homogenized sample of each part of pineapple was subjected to extraction and GC-MS analysis [66].

### 4.3. Sample Preparation and Extraction of Pineapple from Different Ripening Stages 

Pineapple fruit of different ripening stages collected in Indonesia was divided into three parts: Crown, flesh, and peel. Each part was cut into small pieces and placed into a Pyrex tube covered with holed parafilm. Samples were quenched by immersing the Pyrex tubes in liquid nitrogen prior to lyophilization using the VD-800R Freeze dryer (Taitec, Saitama, Japan). Freeze-dried pineapple samples were transported from Indonesia to Japan within a day. The samples were homogenized and ground into a fine powder using Multi-beads shocker. This extraction method was conducted based on the method described in our previous study [66]. Pineapple samples (10 mg), blank samples and quality control (QC) samples were extracted together and lyophilized in a single day. QC samples were prepared by collecting small aliquots of each sample obtained in this study.

All samples were extracted using the mixture of methanol (Wako Chemical, Osaka, Japan), chloroform (Kishida Chemical Co. Ltd, Osaka, Japan), ultrapure water (Wako Chemical, Osaka, Japan); in the ratio of 2.5/1/1 (*v*/*v*/*v*) containing 100 μg/mL ribitol as an internal standard. The mixture was incubated at 37 °C, 1200 rpm for 30 min followed by centrifugation for 3 min at 40 °C. Six hundred microliters of supernatant was transferred to a new 1.5 mL microtube and 300 μL water was added into the mixture. The sample mixture was centrifuged for 3 min at 40 °C and 400 μL of supernatant was transferred to a new microtube and closed with a holed cap. The solvent from the sample mixture was evaporated for 1 h at room temperature followed by lyophilization overnight. All samples were analyzed in triplicates (*n* = 3). One hundred microliters of methoxyamine hydrochloride (20 mg/mL in pyridine) was added into lyophilized samples and incubated in thermomixer for 90 min at 30 °C. Subsequently, 50 μL N-Methyl-N-trimethylsilyl-trifluoroacetamide (MSTFA) (GL Sciences) was added to the samples and incubated for 30 min at 37 °C.

### 4.4. GC-MS Analysis

GC-Q/MS analysis was performed on a GC-MS QP2010 Ultra (Shimadzu, Kyoto, Japan) equipped with an InertCap 5 MS/NP column (GL Sciences). Tuning and calibration of the mass spectrometer were done prior to analysis. One microliter of the derivatized sample was injected in split mode, 25:1 (*v*/*v*), with an injection temperature of 230 °C. The carrier gas (He) flow was 1.12 mL/min with a linear velocity of 39 cm/s. The column temperature was held at 80 °C for 2 min, increased by 15 °C/min to 330 °C, and then held for 6 min. The transfer line and ion source temperatures were 250 and 200 °C, respectively. Ions were generated by electron ionization (EI) at 0.94 kV. Spectra were recorded at 10,000 u/s (check value) over the mass range *m*/*z* 85−500. A standard alkane mixture (C8–C40) was injected prior to analysis for peak identification.

### 4.5. GC-MS Data Analysis

The raw data obtained from the analysis was converted to the AIA file using GCMS solution software package (Shimadzu, Kyoto, Japan). Peak alignment, peak filtering and annotation was conducted by MS-DIAL ver. 4.00 using GCMS-5MP Library (Riken, Kanagawa, Japan). Peak confirmation of important metabolites, namely inositol, mannose, galactose, melezitose were conducted by co-injection with authentic standard (Wako Pure Chemical Industries Ltd., Osaka, Japan; Sigma-Aldrich Japan Ltd., Tokyo, Japan; Alfa Aesar Ltd., Heysham, UK).

### 4.6. Statistical Analysis

Annotated metabolites from GC-MS analysis were pre-treated by normalizing each metabolite peak height to internal standard (ribitol). Normalized data were scaled by autoscaling and without transformation subjected to PCA (Principal Component Analysis) using SIMCA-P+ version 13 (Umetrics, Umea, Sweden). Principal component analysis is an unsupervised analysis that is useful as a dimension-reduction tool in order to easily observe trends, clusters and outliers [25]. Other than PCA, a projections to latent structures (PLS) regression model that is constructed from the maximal correlation of explanatory variable (x-variable) with response variable (y-variable) offers ranking of metabolites correlation with a certain quantitative phenotype [67,68]. In particular, orthogonal projections to latent structures (OPLS) regression model is very useful for reducing many variables to limited latent variables [26]. Parts that show ripening trends in PCA were subjected to OPLS (Orthogonal Projections to Latent Structures) analyses using SIMCA-P+ version 13. From OPLS analyses, variable importance in projection (VIP) were calculated for each metabolite. The top five highest VIP score’s metabolites in each pineapple part were statistically analyzed by analysis of variance (ANOVA) with Tukey’s post hoc test performed using JASP Version 0.11.1 (JASP Team, Amsterdam, Netherlands). The statistical analysis was conducted to evaluate the differences among mean values of VIP metabolites obtained from all ripening stages. Differences were considered significant if *p* < 0.05.

## Supplementary Materials

The following are available online at https://www.mdpi.com/2218-1989/10/4/134/s1, Table S1: Complete list of annotated metabolites with RSD < 20% for sample preparation analysis, Table S2: Complete list of annotated metabolites with RSD < 20% for different part analysis, Table S3: Complete list of annotated metabolites with RSD < 20% for ripening process analysis, Table S4: metabolites with VIP score (more than 1) and its coefficient, Figure S1: Visual experimental design on pineapple ripening study, Figure S2: Venn diagram of annotated metabolites in crown, flesh, and peel part on different parts analysis, Figure S3: Venn diagram of annotated metabolites in crown, flesh, and peel part on pineapple ripening analysis.
