# Supplementary material for: GC-MS Based Metabolite Profiling to Monitor Ripening-Specific Metabolites in Pineapple (Ananas comosus)
Source: Metabolites. 2020 Mar 31;10(4):134. doi: 10.3390/metabo10040134 (PMC7240947; doi:10.3390/metabo10040134)

metabolites

Supplementary Materials

Table S1. Complete list of annotated metabolites with RSD<20% for sample preparation analysis

No	Metabolite	RI*	Quantitative m/z	Annotation
1	Propyleneglycol	1002.62	117	GL-sciences library
2	2-Hydroxypyridine	1038.57	152	GL-sciences library and NIST11
3	Isoleucine	1179.92	86	GL-sciences library and NIST11
4	Serine	1262.86	116	GL-sciences library and NIST11
5	2-Aminoethanol	1275.53	174	GL-sciences library and NIST11
6	Phosphate	1282.02	299	GL-sciences library and NIST11
7	Threonine	1300.99	117	GL-sciences library and NIST11
8	Glycine	1316.35	174	GL-sciences library and NIST11
9	Glyceric acid	1340.02	147	GL-sciences library and NIST11
10	β-Alanine	1436.77	174	GL-sciences library
11	Malic acid	1497.79	147	GL-sciences library and NIST11
12	Meso erythritol	1523.37	217	GL-sciences library and NIST11
13	Pyroglutamic acid	1534.12	156	GL-sciences library
14	4-Aminobutyric acid	1541.43	174	GL-sciences library and NIST11
15	Threonic acid	1576.53	147	GL-sciences library and NIST11
16	Xylose +Lyxose	1671.88	103	GL-sciences library
17	Xylose	1678.62	103	GL-sciences library and NIST11
18	Lyxose	1684.64	103	GL-sciences library and NIST11
19	Ribose	1700.01	103	GL-sciences library and NIST11
20	Putrescine	1757.13	174	GL-sciences library and NIST11
21	Xylonic acid	1793.27	292	GL-sciences library
22	Isocitric acid+Citric acid	1839.77	273	GL-sciences library and NIST11
23	Quinic acid	1890.26	345	GL-sciences library
24	Psicose +Tagatose	1904.94	103	GL-sciences library and NIST11
25	Fructose	1915.11	103	GL-sciences library and NIST11
26	Mannose	1920.06	319	GL-sciences library and NIST11
27	Galactose	1927.03	319	GL-sciences library and NIST11
28	Glucose	1933.51	205	GL-sciences library and NIST11
29	Lysine	1940.06	174	GL-sciences library
30	Galactose+Glucose	1953.11	205	GL-sciences library
31	Tyrosine	1958.30	218	GL-sciences library
32	Sorbitol	1976.31	217	GL-sciences library and NIST11
33	Galacturonic acid	1981.4	333	GL-sciences library

34	Galactitol	1984.89	217	GL-sciences library
35	Gluconic acid	2036.73	292	GL-sciences library
36	Glucarate	2059.15	333	GL-sciences library and NIST11
37	Inositol	2131.83	217	GL-sciences library and NIST11
38	5-Hydroxy tryptamine	2516.82	174	GL-sciences library
39	Sucrose	2709.32	363	GL-sciences library and NIST11
40	β-Lactose	2773.11	217	GL-sciences library and NIST11
41	Trehalose	2814.14	361	GL-sciences library
42	Turanose	2830.42	217	GL-sciences library
43	Maltose	2856.24	361	GL-sciences library and NIST11
44	Gentiobiose	2888.12	204	GL-sciences library and NIST11
45	Maltitol	2936.32	204	GL-sciences library
46	Melibiose	2949.51	204	GL-sciences library
47	Raffinose	3511.31	217	GL-sciences library

*Retention indices (RI) were calculated using standard alkane mixture (C_{10}-C_{40}).

Peak annotation was as follows:

- Comparison of the retention indices (RI) and their mass spectra with GL-science library,
- 80% upwards matching to NIST11 library

Table S2. Complete list of annotated metabolites with RSD<20% for different parts analysis

Crown				
**No**	**Metabolite**	**RI***	**Quantitative m/z**	**Annotation**
1	Alanine	1107.12	116	GL-sciences library and NIST11
2	Valine	1223.16	144	GL-sciences library and NIST11
3	Glyceraldehyde	1224.08	128	GL-sciences library
4	Leucine	1279.57	158	GL-sciences library and NIST11
5	Isoleucine	1301.65	158	GL-sciences library and NIST11
6	Proline	1306.06	142	GL-sciences library and NIST11
7	Serine	1370.36	204	GL-sciences library and NIST11
8	Threonine	1398.02	218	GL-sciences library and NIST11
9	Malic acid	1498.42	147	GL-sciences library and NIST11
10	Threitol	1514.73	103	GL-sciences library
11	Methionine	1530.29	176	GL-sciences library
12	4-Aminobutyric acid	1541.53	174	GL-sciences library and NIST11
13	Threonic acid	1577.10	147	GL-sciences library and NIST11
14	Glutamic acid	1629.40	246	GL-sciences library and NIST11
15	Phenylalanine	1642.13	218	GL-sciences library and NIST11
16	Asparagine	1683.54	116	GL-sciences library and NIST11
17	Arabionose	1685.33	103	GL-sciences library and NIST11
18	Xylulose+Ribulose	1700.41	147	GL-sciences library
19	Ribose	1700.79	103	GL-sciences library and NIST11
20	Rhamnose	1745.06	117	GL-sciences library and NIST11
21	Arabitol	1746.41	217	GL-sciences library
22	Putrescine	1757.17	174	GL-sciences library and NIST11
23	Glutamine	1785.64	156	GL-sciences library
24	Xylonic acid	1793.58	292	GL-sciences library
25	Shikimic acid	1821.25	204	GL-sciences library
26	Isocitric acid+Citric acid	1840.79	273	GL-sciences library and NIST11
27	Adenine	1884.76	264	GL-sciences library
28	Quinic acid	1891.18	345	GL-sciences library
29	Psicose +Tagatose	1905.71	306	GL-sciences library and NIST11
30	Sorbose	1906.61	103	GL-sciences library and NIST11
31	Fructose	1816.49	103	GL-sciences library and NIST11
32	Galactose	1927.86	205	GL-sciences library and NIST11
33	Tyramine	1932.78	174	GL-sciences library
34	Glucose	1935.59	319	GL-sciences library and NIST11

35	Allose +Mannose	1936.33	319	GL-sciences library
36	Lysine	1941.06	174	GL-sciences library and NIST11
37	Mannitol	1968.32	319	GL-sciences library and NIST11
38	Sorbitol	1975.83	147	GL-sciences library and NIST11
39	Galacturonic acid	1982.28	333	GL-sciences library
40	Glucarate	2003.34	147	GL-sciences library and NIST11
41	Gluconic acid	2039.82	333	GL-sciences library
42	Inositol	2132.12	217	GL-sciences library and NIST11
43	Guanine	2146.03	352	GL-sciences library
44	Tryptophan	2217.38	218	GL-sciences library
45	Spermidine	2284.89	144	GL-sciences library
46	Fructose 6-phosphate	2357.09	315	GL-sciences library
47	Sucrose	2707.26	361	GL-sciences library and NIST11
48	β-Lactose	2780.38	117	GL-sciences library and NIST11
49	Trehalose	2816.61	361	GL-sciences library
50	Lactitol	2840.38	361	GL-sciences library
51	Gentiobiose	2890.84	204	GL-sciences library and NIST11
52	Maltitol	2950.89	204	GL-sciences library
53	Galactinol	3076.88	204	GL-sciences library
54	Raffinose	3503.75	361	GL-sciences library
		**Flesh**		
**No**	**Metabolite**	**RI***	**Quantitative m/z**	**Annotation**
1	Propyleneglycol	1002.9	117	GL-sciences library
2	Alanine	1107.12	116	GL-sciences library and NIST11
3	Valine	1223.16	144	GL-sciences library and NIST11
4	Proline	1306.06	142	GL-sciences library and NIST11
5	Serine	1370.36	204	GL-sciences library and NIST11
6	Malic acid	1498.42	147	GL-sciences library and NIST11
7	Methionine	1530.29	176	GL-sciences library
8	4-Aminobutyric acid	1541.53	174	GL-sciences library and NIST11
9	Threonic acid	1577.10	147	GL-sciences library
10	Xylose +Lyxose	1672.56	103	GL-sciences library
11	Lyxose	1679.35	103	GL-sciences library and NIST11
12	Asparagine	1683.54	116	GL-sciences library and NIST11
13	Arabionose	1685.33	103	GL-sciences library and NIST11
14	Xylulose+Ribulose	1700.41	147	GL-sciences library
15	Ribose	1700.79	103	GL-sciences library and NIST11

16	Xylonic acid	1793.58	292	GL-sciences library
17	Shikimic acid	1821.25	204	GL-sciences library
18	Isocitric acid+Citric acid	1840.79	273	GL-sciences library and NIST11
19	Quinic acid	1891.18	345	GL-sciences library
20	Psicose +Tagatose	1905.71	306	GL-sciences library and NIST11
21	Sorbose	1906.61	103	GL-sciences library and NIST11
22	Fructose	1816.49	103	GL-sciences library and NIST11
23	Mannose	1921.10	319	GL-sciences library and NIST11
24	Galactose	1927.86	205	GL-sciences library and NIST11
25	Tyramine	1932.78	174	GL-sciences library
26	Glucose	1935.59	319	GL-sciences library and NIST11
27	Allose +Mannose	1936.33	319	GL-sciences library
28	Galactose+Glucose	1954.48	319	GL-sciences library
29	Sorbitol	1975.83	147	GL-sciences library and NIST11
30	Glucarate	2003.34	147	GL-sciences library and NIST11
31	Gluconic acid	2039.82	333	GL-sciences library
32	5-Hydroxy tryptamine	2516.25	174	GL-sciences library
33	Sucrose	2707.26	361	GL-sciences library and NIST11
34	β-Lactose	2780.38	117	GL-sciences library and NIST11
35	Trehalose	2816.61	361	GL-sciences library
36	Lactitol	2840.38	361	GL-sciences library
37	Maltose	2858.24	361	GL-sciences library and NIST11
38	Maltitol	2950.89	204	GL-sciences library
39	Melibiose	2950.89	204	GL-sciences library
40	Galactinol	3076.88	204	GL-sciences library
41	Raffinose	3503.75	361	GL-sciences library
42	Paeoniflorin	3514.07	217	GL-sciences library
43	Guanine	2146.03	352	GL-sciences library and NIST11
44	Inositol	2132.12	217	GL-sciences library and NIST11
		**Peel**		
**No**	**Metabolite**	**RI***	**Quantitative m/z**	**Annotation**
1	Propyleneglycol	1002.9	117	GL-sciences library
2	Alanine	1107.12	116	GL-sciences library and NIST11
3	Valine	1223.16	144	GL-sciences library and NIST11
4	Proline	1306.06	142	GL-sciences library and NIST11
5	Serine	1370.36	204	GL-sciences library and NIST11
6	Malic acid	1498.42	147	GL-sciences library and NIST11

7	Methionine	1530.29	176	GL-sciences library
8	4-Aminobutyric acid	1541.53	174	GL-sciences library and NIST11
9	Threonic acid	1577.10	147	GL-sciences library
10	Xylose +Lyxose	1672.56	103	GL-sciences library
11	Lyxose	1679.35	103	GL-sciences library and NIST11
12	Asparagine	1683.54	116	GL-sciences library and NIST11
13	Arabionose	1685.33	103	GL-sciences library and NIST11
14	Xylulose+Ribulose	1700.41	147	GL-sciences library
15	Ribose	1700.79	103	GL-sciences library and NIST11
16	Rhamnose	1745.06	117	GL-sciences library and NIST11
17	Arabitol	1746.41	217	GL-sciences library
18	Glutamine	1785.64	156	GL-sciences library
19	Xylonic acid	1793.58	292	GL-sciences library
20	Shikimic acid	1821.25	204	GL-sciences library
21	Isocitric acid+Citric acid	1840.79	273	GL-sciences library and NIST11
22	Quinic acid	1891.18	345	GL-sciences library
23	Psicose +Tagatose	1905.71	306	GL-sciences library
24	Sorbose	1906.61	103	GL-sciences library and NIST11
25	Fructose	1816.49	103	GL-sciences library and NIST11
26	Mannose	1921.10	319	GL-sciences library and NIST11
27	Galactose	1927.86	205	GL-sciences library and NIST11
28	Tyramine	1932.78	174	GL-sciences library
29	Glucose	1935.59	319	GL-sciences library and NIST11
30	Allose +Mannose	1936.33	319	GL-sciences library
31	Galactose+Glucose	1954.48	319	GL-sciences library
32	Mannitol	1968.32	319	GL-sciences library and NIST11
33	Sorbitol	1975.83	147	GL-sciences library and NIST11
34	Galacturonic acid	1982.28	333	GL-sciences library
35	Glucarate	2003.34	147	GL-sciences library and NIST11
36	Gluconic acid	2039.82	333	GL-sciences library
37	Inositol	2132.12	217	GL-sciences library and NIST11
38	Guanine	2146.03	352	GL-sciences library and NIST11
39	Fructose 6-phosphate	2357.09	315	GL-sciences library
40	5-Hydroxy tryptamine	2516.25	174	GL-sciences library
41	Sucrose	2707.26	361	GL-sciences library and NIST11
42	β-Lactose	2780.38	117	GL-sciences library and NIST11
43	Trehalose	2816.61	361	GL-sciences library
44	Lactitol	2840.38	361	GL-sciences library

45	Maltose	2858.24	361	GL-sciences library and NIST11
46	Gentiobiose	2890.84	204	GL-sciences library and NIST11
47	Maltitol	2950.89	204	GL-sciences library
48	Melibiose	2950.89	204	GL-sciences library
49	Galactinol	3076.88	204	GL-sciences library
50	Raffinose	3503.75	361	GL-sciences library

*Retention indices (RI) were calculated using standard alkane mixture (C_{10}-C_{40}).

Peak annotation was as follows:

- Comparison of the retention indices (RI) and their mass spectra with GL-science library,
- 80% upwards matching to NIST11 library

Table S3. Complete list of annotated metabolites with RSD<20% for ripening process analysis

* = metabolites that only present in either crown, flesh, or peel part

Crown				
**No**	**Metabolite**	**RI***	**Quantitative m/z**	**Annotation**
1	2-Aminoethanol	1275.61	174	GL-sciences library and NIST11
2	2-Hydroxypyridine	1038.83	152	GL-sciences library and NIST11
3	4-Aminobutyric acid	1541.90	174	GL-sciences library and NIST11
4	Alanine	1107.02	116	GL-sciences library and NIST11
5	Arabionose	1685.38	103	GL-sciences library and NIST11
6	Ascorbic acid*	1975.45	319	GL-sciences library
7	Asparagine	1683.48	116	GL-sciences library and NIST11
8	β-Lactose	2773.80	204	GL-sciences library and NIST11
9	Chlorogenic acid*	3169.11	345	GL-sciences library
10	Fructose 6-phosphate	2371.14	387	GL-sciences library
11	Fructose	1915.19	103	GL-sciences library and NIST11
12	Galactose	1927.13	309	GL-sciences library and NIST11
13	Galactose+Glucose	1953.42	319	GL-sciences library
14	Galacturonic acid	1981.79	333	GL-sciences library
15	Gentiobiose	2889.32	204	GL-sciences library and NIST11
16	Glucarate	2059.52	333	GL-sciences library and NIST11
17	Glucose	1933.45	319	GL-sciences library and NIST11
18	Glutamic acid	1629.50	246	GL-sciences library and NIST11
19	Glutamine*	1785.77	156	GL-sciences library
20	Glyceric acid	1340.15	147	GL-sciences library and NIST11
21	Glycine	1316.47	174	GL-sciences library and NIST11
22	Inositol	2132.57	217	GL-sciences library and NIST11
23	Isocitric acid+Citric acid	1841.85	273	GL-sciences library and NIST11
24	Isoleucine	1301.52	158	GL-sciences library and NIST11
25	Leucine*	1279.71	158	GL-sciences library and NIST11
26	Lysine	1940.35	174	GL-sciences library
27	Lyxose	1685.35	103	GL-sciences library and NIST11
28	Malic acid	1498.61	147	GL-sciences library and NIST11
29	Maltitol	2936.87	204	GL-sciences library
30	Mannitol	1967.73	319	GL-sciences library and NIST11
31	Melibiose	2949.37	361	GL-sciences library
32	Meso erythritol	1523.81	147	GL-sciences library and NIST11
33	N-Acetyl glucosamine*	2118.52	147	GL-sciences library and NIST11

34	Oxalacetic acid+Pyruvate	1050.97	174	GL-sciences library
35	Phenylalanine	1642.44	218	GL-sciences library and NIST11
36	Phosphate	1282.05	299	GL-sciences library and NIST11
37	Proline	1306.0	142	GL-sciences library and NIST11
38	Psicose+Tagatose	1905.19	103	GL-sciences library and NIST11
39	Putrescine*	1757.65	174	GL-sciences library and NIST11
40	Quinic acid	1890.97	345	GL-sciences library
41	Raffinose	3500.23	361	GL-sciences library
42	Rhamnose	1744.84	117	GL-sciences library and NIST11
43	Ribitol	1752.37	217	GL-sciences library, NIST11, and STD
44	Ribose	1700.54	103	GL-sciences library and NIST11
45	Serine	1262.93	116	GL-sciences library and NIST11
46	Sucrose	2704.68	361	GL-sciences library and NIST11
47	Threitol*	1515.11	217	GL-sciences library
48	Threonic acid	1577.02	147	GL-sciences library and NIST11
49	Threonine	1397.86	218	GL-sciences library and NIST11
50	Trehalose	2814.93	361	GL-sciences library
51	Tryptophan	2219.10	218	GL-sciences library
52	Tyrosine	1958.71	218	GL-sciences library
53	Uric acid*	2127.02	441	GL-sciences library*
54	Valine	1223.4	144	GL-sciences library and NIST11
55	Xylonic acid	1793.15	292	GL-sciences library
56	Xylose	1679.15	103	GL-sciences library and NIST11
**Flesh**				
**No**	**Metabolite**	**RI***	**Quantitative m/z**	**Annotation**
1	2-Aminoethanol	1275.39	174	GL-sciences library and NIST11
2	4-Aminobutyric acid	1541.37	174	GL-sciences library and NIST11
3	5-Hydroxy tryptamine*	2517.11	174	GL-sciences library
4	Alanine	1371.36	188	GL-sciences library and NIST11
5	Aspartic acid	1530.54	232	GL-sciences library
6	β -Lactose	2773.87	204	GL-sciences library and NIST11
7	Fructose	1915.21	103	GL-sciences library and NIST11
8	Galactinol	3075.23	204	GL-sciences library
9	Galactose	1927.03	319	GL-sciences library and NIST11
10	Galactose+Glucose	1953.44	319	GL-sciences library
11	Galacturonic acid	1981.75	147	GL-sciences library

12	Gentiobiose	2888.76	204	GL-sciences library and NIST11
13	Glucarate	2058.66	204	GL-sciences library and NIST11
14	Gluconic acid	2035.99	147	GL-sciences library
15	Glucose	1933.95	319	GL-sciences library and NIST11
16	Glutamic acid	1629.04	246	GL-sciences library and NIST11
17	Glycine	1316.22	174	GL-sciences library and NIST11
18	Inositol	2131.83	217	GL-sciences library, NIST11, and STD
19	Isocitric acid+Citric acid	1840.31	273	GL-sciences library and NIST11
20	Lysine	1939.74	174	GL-sciences library
21	Lyxose	1684.88	103	GL-sciences library and NIST11
22	Malic acid	1497.61	147	GL-sciences library and NIST11
23	Maltitol	2936.84	204	GL-sciences library
24	Maltose	2856.61	361	GL-sciences library and NIST11
25	Melezitose*	3590.08	361	GL-sciences library and STD
26	Melibiose	2949.63	361	GL-sciences library
27	Oxalacetic acid+Pyruvate	1050.55	174	GL-sciences library
28	Phosphate	1281.72	299	GL-sciences library and NIST11
29	Proline	1305.97	142	GL-sciences library and NIST11
30	Psicose+Tagatose	1905.13	103	GL-sciences library and NIST11
31	Pyroglutamic acid	1534.04	156	GL-sciences library
32	Quinic acid	1890.39	345	GL-sciences library and STD
33	Raffinose	3499.49	361	GL-sciences library
34	Rhamnose	1744.43	117	GL-sciences library and NIST11
35	Ribitol	1751.91	217	GL-sciences library, NIST11, and STD
36	Serine	1369.89	204	GL-sciences library and NIST11
37	Sorbitol	1975.10	319	GL-sciences library and NIST11
38	Spermidine*	2284.89	368	GL-sciences library
39	Sucrose	2708.22	362	GL-sciences library and NIST11
40	Threonic acid	1576.48	292	GL-sciences library and NIST11
41	Trehalose	2815.30	361	GL-sciences library
42	Turanose	2830.75	217	GL-sciences library
43	Tyrosine	1958.97	218	GL-sciences library
44	Valine	1223.06	144	GL-sciences library and NIST11
45	Xylonic acid	1802.68	103	GL-sciences library
46	Xylose+Lyxose	1671.96	103	GL-sciences library
47	Xylose	1678.61	103	GL-sciences library and NIST11
**Peel**				

No	Metabolite	RI*	Quantitative m/z	Annotation
1	1,6-Anhydroglucose*	1726.19	204	GL-sciences library
2	2-Aminoethanol	1275.80	174	GL-sciences library and NIST11
3	2-Hydroxypyridine	1038.98	152	GL-sciences library and NIST11
4	4-Aminobutyric acid	1541.83	174	GL-sciences library and NIST11
5	α-Ketoglutaric acid*	1583.58	147	GL-sciences library
6	Alanine	1371.86	188	GL-sciences library and NIST11
7	Allose+Mannose	1936.41	273	GL-sciences library and NIST11
8	Arabionose	1685.53	103	GL-sciences library and NIST11
9	Aspartic acid	1531.03	232	GL-sciences library
10	β -Lactose	2774.12	204	GL-sciences library and NIST11
11	Fructose 6-phosphate	2371.23	387	GL-sciences library
12	Fructose	1915.69	103	GL-sciences library and NIST11
13	Galactinol	3075.95	204	GL-sciences library
14	Galactose	1927.53	319	GL-sciences library, NIST11, and STD
15	Galactose+Glucose	1953.92	319	GL-sciences library
16	Gentiobiose	2889.44	204	GL-sciences library and NIST11
17	Glucarate	2059.65	333	GL-sciences library and NIST11
18	Gluconic acid	2003.61	147	GL-sciences library
19	Glucose	1934.39	319	GL-sciences library, NIST11, and STD
20	Glutamic acid	1629.56	246	GL-sciences library and NIST11
21	Glyceric acid	1340.31	189	GL-sciences library
22	Glycine	1316.38	147	GL-sciences library and NIST11
23	Inositol	2132.77	217	GL-sciences library, NIST11, and STD
24	Isocitric acid+Citric acid	1840.73	273	GL-sciences library and NIST11
25	Isoleucine	1301.68	158	GL-sciences library and NIST11
26	Lyxose	1679.11	103	GL-sciences library and NIST11
27	Malic acid	1498.09	147	GL-sciences library and NIST11
28	Maltitol	2937.58	204	GL-sciences library
29	Maltose	2853.17	319	GL-sciences library and NIST11
30	Mannitol	1967.98	319	GL-sciences library and NIST11
31	Mannose*	1920.82	160	GL-sciences library, NIST11, and STD
32	Melibiose	2950.27	361	GL-sciences library
33	Meso erythritol	1523.84	217	GL-sciences library
34	Oxalacetic acid+Pyruvate	1051.11	174	GL-sciences library
35	Phenylalanine	1642.55	218	GL-sciences library and NIST11

36	Phosphate	1282.15	299	GL-sciences library and NIST11
37	Pyroglutamic acid	1534.56	156	GL-sciences library
38	Quinic acid	1890.97	345	GL-sciences library
39	Raffinose	3500.43	361	GL-sciences library
40	Rhamnose	1744.97	117	GL-sciences library and NIST11
41	Ribitol	1752.38	217	GL-sciences library, NIST11, and STD
42	Ribose	1700.52	103	GL-sciences library
43	Serine	1370.24	204	GL-sciences library and NIST11
44	Sinapinic acid*	2256.58	368	GL-sciences library
45	Sorbitol	1975.97	147	GL-sciences library and NIST11
46	Sorbose*	1905.65	103	GL-sciences library and NIST11
47	Sucrose	2707.94	362	GL-sciences library and NIST11
48	Threonic acid	1577.04	147	GL-sciences library and NIST11
49	Threonine	1398.00	218	GL-sciences library and NIST11
50	Trehalose	2815.29	361	GL-sciences library
51	Tyrosine	1958.86	218	GL-sciences library
52	Valine	1223.59	144	GL-sciences library and NIST11
53	Xylonic acid	1782.37	103	GL-sciences library
54	Xylose+Lyxose	1672.39	103	GL-sciences library

*Retention indices (RI) were calculated using standard alkane mixture (C_{10}-C_{40}).

Peak annotation was as follows:

- Comparison of the retention indices (RI) and their mass spectra with GL-science library,
- 80% upwards matching to NIST11 library
- Co-injection with authentic standard by spiking into extracted samples.

Table S4. Metabolites with VIP score (more than 1) and its coefficients

Flesh				Peel			
No	Metabolite	VIP Score	Coefficients	No	Metabolite	VIP Score	Coefficients
1	Melezitose	1.805	0.086	1	Inositol	1.858	-0.088
2	Inositol	1.784	-0.085	2	Mannose	1.712	-0.066
3	Xylonic acid	1.736	0.075	3	Galactose	1.700	-0.053
4	Gluconic acid	1.689	0.069	4	Sucrose	1.661	0.079
5	Raffinose	1.687	-0.070	5	Aspartic acid	1.627	-0.066
6	Threonic acid	1.617	0.090	6	Galactose+Glucose	1.596	-0.065
7	β-Lactose	1.553	-0.076	7	1,6-Anhydroglucose	1.535	0.071
8	Quinic acid	1.457	-0.058	8	Tyrosine	1.423	-0.044
9	Oxalacetic acid+Pyruvate	1.378	-0.054	9	Allose+Mannose	1.377	-0.036
10	Melibiose	1.373	0.042	10	Melibiose	1.360	-0.050
11	Malic acid	1.248	-0.057	11	4-Aminobutyric acid	1.294	-0.036
12	Galactose	1.241	-0.048	12	Glucose	1.292	-0.023
13	Maltose	1.203	0.033	13	Pyroglutamic acid	1.267	-0.031
14	Turanose	1.200	0.046	14	Meso erythritol	1.247	0.036
15	Galacturonic acid	1.145	-0.106	15	Raffinose	1.230	-0.025
16	2-Aminoethanol	1.019	0.013	16	Galactinol	1.216	-0.043
17	Gentiobiose	1.004	0.058	17	Fructose	1.215	-0.027
				18	Quinic acid	1.205	-0.055
				19	Xylose+Lyxose	1.198	0.090
				20	Lyxose	1.196	0.015
				21	Glutamic acid	1.155	-0.043
				22	Threonic acid	1.067	-0.034
				23	Gentiobiose	1.032	0.038
				24	Glucarate	1.004	-0.041

Figure S1. Visual experimental design on pineapple ripening study in this manuscript

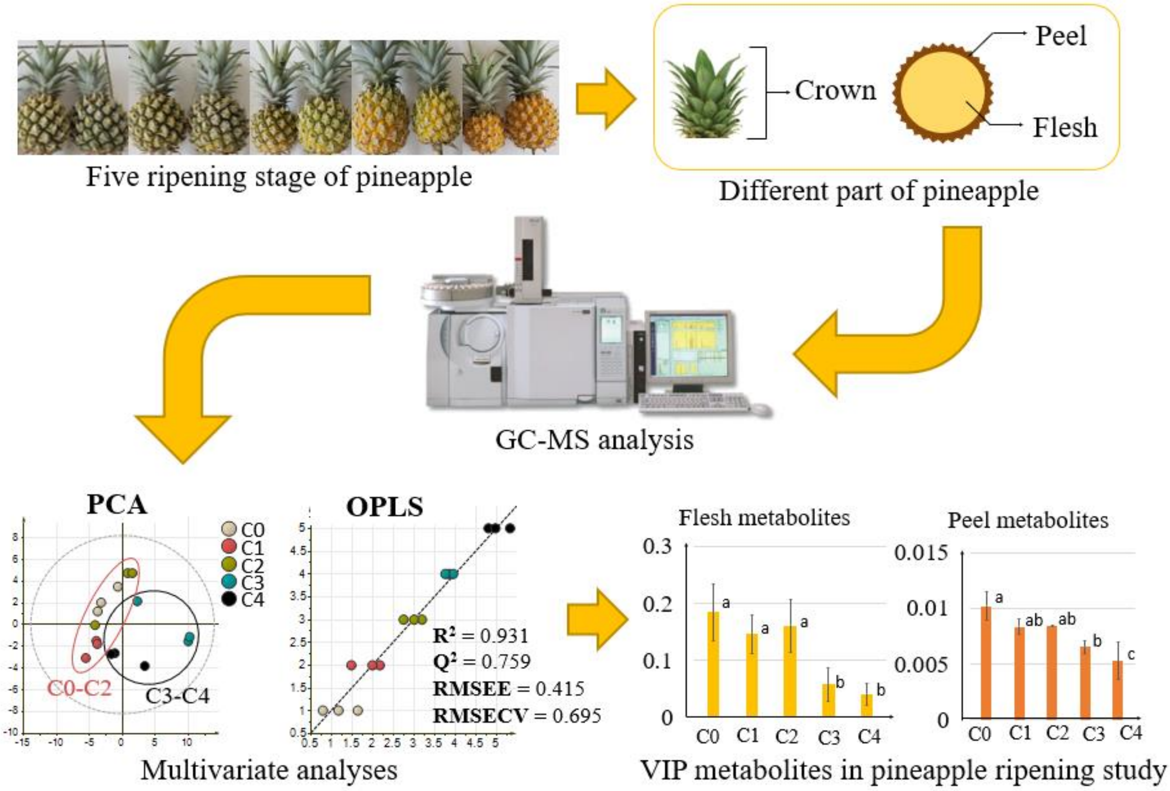

Figure S2. Venn diagram of annotated metabolites in crown, flesh, and peel part on different parts analysis

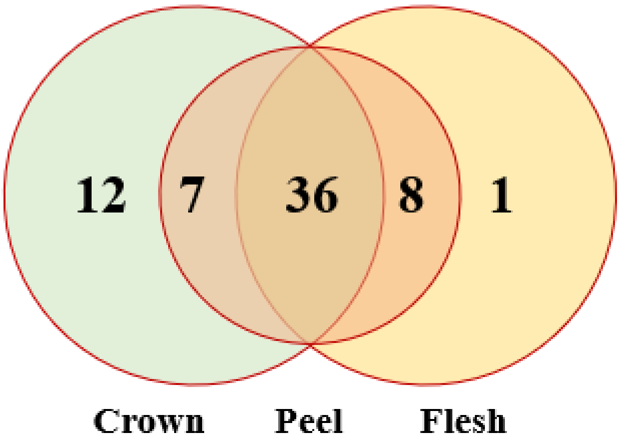

Figure S3. Venn diagram of annotated metabolites in crown, flesh, and peel part on pineapple ripening analysis

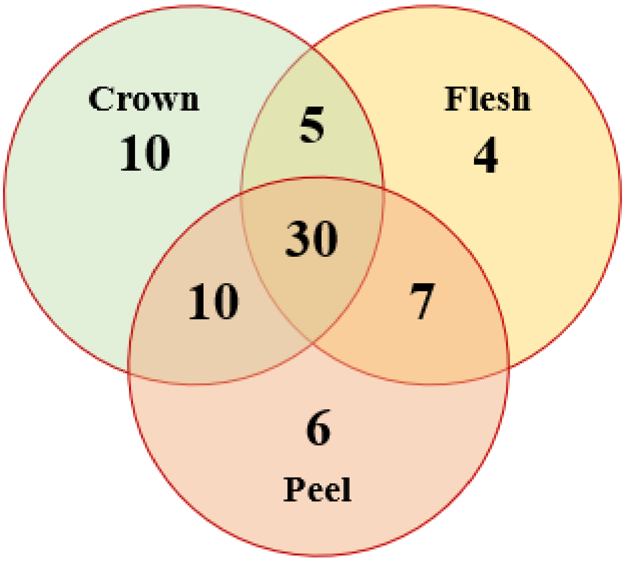

Supplement: Supplementary file 1 [file metabolites-10-00134-s001.pdf]
